# Longitudinal thalamic white and grey matter changes associated with visual hallucinations in Parkinson’s disease

**DOI:** 10.1136/jnnp-2021-326630

**Published:** 2021-09-28

**Authors:** Angeliki Zarkali, Peter McColgan, Louise Ann Leyland, Andrew John Lees, Rimona Sharon Weil

**Affiliations:** 1 Dementia Research Centre, University College London, London, UK; 2 Huntington’s Disease Centre, UCL Institute of Neurology, London, UK; 3 Reta Lila Weston Institute, Institute of Neurology, London, UK; 4 Wellcome Centre for Human Neuroimaging, University College London, London, UK; 5 Movement Disorders Consortium, National Hospital for Neurology and Neurosurgery, London, UK

**Keywords:** Parkinson's disease, hallucinations

## Abstract

**Objective:**

Visual hallucinations are common in Parkinson’s disease (PD) and associated with worse outcomes. Large-scale network imbalance is seen in PD-associated hallucinations, but mechanisms remain unclear. As the thalamus is critical in controlling cortical networks, structural thalamic changes could underlie network dysfunction in PD hallucinations.

**Methods:**

We used whole-brain fixel-based analysis and cortical thickness measures to examine longitudinal white and grey matter changes in 76 patients with PD (15 hallucinators, 61 non-hallucinators) and 26 controls at baseline, and after 18 months. We compared white matter and cortical thickness, adjusting for age, gender, time-between-scans and intracranial volume. To assess thalamic changes, we extracted volumes for 50 thalamic subnuclei (25 each hemisphere) and mean fibre cross-section (FC) for white matter tracts originating in each subnucleus and examined longitudinal change in PD-hallucinators versus non-hallucinators.

**Results:**

PD hallucinators showed white matter changes within the corpus callosum at baseline and extensive posterior tract involvement over time. Less extensive cortical thickness changes were only seen after follow-up. White matter connections from the right medial mediodorsal magnocellular thalamic nucleus showed reduced FC in PD hallucinators at baseline followed by volume reductions longitudinally. After follow-up, almost all thalamic subnuclei showed tract losses in PD hallucinators compared with non-hallucinators.

**Interpretation:**

PD hallucinators show white matter loss particularly in posterior connections and in thalamic nuclei, over time with relatively preserved cortical thickness. The right medial mediodorsal thalamic nucleus shows both connectivity and volume loss in PD hallucinations. Our findings provide mechanistic insights into the drivers of network imbalance in PD hallucinations and potential therapeutic targets.

## Introduction

Visual hallucinations (VH) are common in Parkinson’s disease (PD), can cause significant distress to affected individuals and their families and are associated with worse outcomes:^1^ patients with PD and VH have worse quality of life,^2^ increased mortality,^3^ higher rates of subsequent dementia^4^ and are more likely to require nursing home care.^5^

PD-associated hallucinations are accompanied by macroscale brain network imbalance with aberrant activation of the default mode network and reduced activity of other networks such as the dorsal attentional network.^6^ Network changes are thought to underlie the impaired accumulation of sensory evidence^7^ and the overweighting of previously held beliefs (at the expense of sensory information)^8^ seen in PD hallucinators. Widespread changes in brain structure are seen in PD hallucinations with loss of grey matter volume across regions including the precuneus, cingulate and superior and inferior frontal gyri^9^ and white matter structure within posterior tracts and at whole-network level.^10 11^ However, our understanding of the drivers of these large-scale network changes remains unclear.

We recently showed that structural connectivity loss may preferentially affect areas that normally exert high levels of influence over the whole-brain network and are particularly important for switching the brain between states.^12^ The thalamus, a connection-rich diencephalic hub critical for cortical sensory filtering,^13 14^ has been recently proposed as a potential key driver for unbalanced network activation.^15 16^ Thalamic hypometabolism and atrophy are seen in patients with PD and hallucinations^17^ and is also present in frontotemporal dementia associated with C9orf72 mutations where hallucinations are reported.^18^ Reduced thalamic connectivity with the prefrontal cortex (PFC) is also seen in relation to hallucinations in psychosis.^19^ However, the thalamus is a heterogeneous structure comprised of distinct nuclei with different cortical projections and functions.^13^ Specific thalamic subnuclei may be implicated in Parkinson’s hallucinations and can now be robustly segmented using a recently described probabilistic atlas.^20^

White matter changes, detected using diffusion-weighted MRI, may be more sensitive to early degenerative processes in PD than grey matter loss, as they reflect changes in axons rather than neuronal loss.^21^ White matter changes may occur at an earlier stage in PD:^22^ in whole-brain studies of PD and cognition, white matter loss is seen before significant grey matter atrophy.^23 24^

Here, we attempt to clarify the longitudinal grey and white matter changes underlying VH in PD and assess the relative involvement of different thalamic nuclei. We examined whole-brain cortical thickness and white matter integrity using fixel-based analysis, a sensitive and fibre-specific framework,^25^ at baseline and after 18 months in patients with PD with (PD-VH) and without hallucinations (PD non-VH). Additionally, we assessed changes in grey matter volume of 50 thalamic subnuclei and macrostructural white matter integrity of their respective corticothalamic projections at baseline and longitudinally. We hypothesised that corticothalamic connection loss would precede volume loss in PD-VH and that subregions of the thalamus would show differential vulnerability to degeneration.

## Methods

### Participants

Participants were recruited to the National Hospital, Queen Square and underwent clinical assessments and brain imaging at baseline and after 18 months (visit 2). Only participants who had structural and diffusion-weighted imaging satisfying predetermined quality control criteria at both visits were included (see online supplemental methods for details on excluded participants). 101 participants were included: 76 patients with PD and 26 age-matched controls (from spouses and volunteer databases). All patients with PD satisfied the Queen Square Brain Bank criteria.^26^ The study was approved by the Queen Square ethics committee (15/LO/00476) and all participants provided written informed consent prior to taking part.

10.1136/jnnp-2021-326630.supp1 Supplementary data

Participants with PD were classified as PD with VH (PD-VH, n=15) if they scored more than 1 for question 2 of the Unified Parkinson’s Disease Rating Scale (UPDRS): ‘Over the past week have you seen, heard, smelled or felt things that were not really there?’ in either study visit; all participants reported hallucinations in the visual domain. All other participants were classified as PD non-VH (n=61). Further details on the frequency and severity of VH were collected for all participants using the University of Miami Parkinson’s Disease Hallucinations Questionnaire (UM-PDHQ).^27^

All participants underwent assessments of general cognition using the Mini-Mental State Examination (MMSE) and Montreal Cognitive Assessment (MoCA). Comprehensive domain-specific cognitive assessments were also performed using two tests per cognitive domain (see online supplemental methods for details). Levodopa dose equivalence scores were calculated for PD participants.^28^

### MRI data acquisition

All MRI data were acquired on the same 3T Siemens Magnetom Prisma scanner (Siemens) with a 64-channel head coil. Magnetisation prepared rapid acquisition gradient echo was acquired using the following parameters: 1×1×1 mm voxel, TE=3.34 ms, TR=2530 ms, flip angle=7°. Diffusion-weighted imaging (DWI) was acquired using these parameters: b=50 s/mm^2^/17 directions, b=300 s/mm^2^/8 directions, b=1000 s/mm^2^/64 directions, b=2000 s/mm^2^/64 directions, 2×2×2 mm isotropic voxels, Echo time (TE)=3260 ms, Repetition time (TR)=58 ms, 72 slices, 2 mm thickness, acceleration factor=2.

### Grey matter imaging processing

FreeSurfer V.6.0 was used with default parameters for cross-sectional processing, then images were automatically processed with the longitudinal stream.^29^ Specifically, an unbiased within-subject template space and image were created using inverse consistent registration. Subsequent processing steps were initialised with common information from the within-subject template, increasing accuracy and statistical power.^29^ After longitudinal processing, surface reconstructions of the template and of images at T1 and T2 were inspected, corrected and reprocessed where necessary.

In addition, thalamic subnuclei volumes were derived from each longitudinally processed Freesurfer reconstruction using a recently described Bayesian segmentation method based on a probabilistic histology-derived atlas.^20^ Volumes were derived for 25 subnuclei for each thalamus: anteroventral, laterodorsal, lateral posterior, ventral anterior, ventral anterior magnocellular, ventral lateral anterior, ventral lateral posterior, ventral posterolateral, ventromedial (VM), central medial (CeM), central lateral, paracentral (Pc), Centromedian, parafascicular, paratenial, Reuniens medial ventral, mediodorsal medial magnocellular (MDm), mediodorsal medial parvocellular, lateral geniculate (LGN), medial geniculate, limitans, pulvinar anterior, pulvinar medial, pulvinar lateral and pulvinar inferior.

### DWI processing

DWI images passing quality control underwent denoising,^30^ removal of ringing artefacts,^31^ eddy-current and motion correction^32^ and bias-field correction.^33^ Spatial resolution was then up sampled to 1.3 mm^3^ voxel size as recommended for fixel-based analysis and intensity normalisation performed across subjects. For each participant, fibre-orientation distributions (FODs) were then computed using multishell three-tissue constrained spherical deconvolution with the group-average response function for each tissue type (grey matter, white matter, cerebrospinal fluid (CSF)).^34^

To allow longitudinal comparison, we created a group-averaged FOD template at baseline from 30 randomly selected subjects (20 PD, 10 controls). Each participant’s FOD was registered to the template^35^ and fixel-based metrics derived: (a) *Fibre density (FD*): a metric of microstructural changes within tracts, (b) *Fibre cross-section (FC):* a relative measure of macrostructural changes and (c) *Combined measure of fibre density and cross-section (FDC*): a combined metric calculated as FD multiplied by FC for each fixel and representing overall white matter integrity.^21^ All preprocessing and analyses of DWI data were performed in MRtrix V.3.0.

To specifically assess the integrity of thalamic connections, we also generated specific tracts per hemisphere from each of the 50 thalamic subnuclei. Each subnucleus was registered to the population template using linear registration with NiftyReg.^36^ Subsequently, a tractogram for each thalamic subnucleus was generated using probabilistic tractography on the population template.^37^ Streamlines were initiated in each thalamic subnucleus to the ipsilateral hemisphere, with the rest of the thalamus excluded to minimise overlap between tracts. This resulted in a single tract-of-interest from each thalamic subnucleus to the cortex. Mean FC was then calculated across each tract-of-interest per participant; FC was chosen as prior works showed it is the most sensitive fibre-specific metric in PD.^10 38^

### Statistical analysis

#### Demographics

Group differences in demographics and clinical characteristics were assessed using independent t-samples and analysis of variance (ANOVA)s for normally distributed continuous (post-hoc Tukey), Mann-Whitney and Kruskall-Wallis for non-normally distributed (post-hoc Dunn) and χ^2^ for categorical variables; statistical significance p<0.05. Shapiro-Wilk was used to assess normality.

#### Whole-brain fixel-based analysis

Non-parametric permutation testing and connectivity-based fixel enhancement (CFE)^25^ was used to identify significant differences in fixel-based metrics. We generated a tractogram with 20 million streamlines using whole-brain probabilistic tractography on the population FOD template; this was filtered to 2 million streamlines using SIFT (spherical deconvolution informed filtering of tractograms).^39^ CFE was performed on the resulting streamlines using the default smoothing parameters (C=0.5, E=2, H=3), with 5000 permutations and family-wise error correction (FWE) for multiple comparisons. FWE-corrected p<0.05 with cluster-extent-based threshold of 10 voxels was considered statistically significant. We used the John Hopkins University atlas to identify white matter fixels across the whole brain for subsequent statistical comparisons, in keeping with previous studies.^10 38 40^ Whole white-matter comparisons were performed at baseline between PD-VH and PD non-VH, using age, gender and intracranial volume as covariates. To implement a longitudinal design matrix, we subtracted each baseline image from the visit 2 image. Whole white-matter statistical analyses were then performed on these difference images with baseline age, gender, intracranial volume and time between scans as covariates.

#### Whole-brain cortical thickness analysis

To determine differences in cortical thickness trajectories over time between PD-VH and PD non-VH, we used Linear Mixed Effect models in MATLAB (The MathWorks) designed for longitudinal FreeSurfer data.^41^ A spatiotemporal novel mass-univariate analysis was performed with cortical thickness as the dependent variable and a random intercept defining subject as a random factor. Additional regressors included the time-between-scans in years (baseline imaging marked as 0), age at baseline, gender, group (PD-VH vs PD non-VH), and group-by-time interaction (variable of interest). Significance maps for group-by-time interactions were corrected for multiple comparisons using a false discovery rate (FDR) correction combined over the left and right hemispheres and saved for later visualisation in freeview.

#### Thalamic subnucleus and tract-of-interest analysis

Thalamic volumes and mean tract FC at baseline were compared between PD-VH and PD non-VH using a linear mixed model with age, gender and intracranial volumes as covariates. To assess differences in longitudinal rate of change for each thalamic subnucleus and each tract-of-interest, respectively, we used a linear mixed model with group-by-time interaction as the variable of interest and group (PD-VH vs PD non-VH), age, gender, and time-between-scans as regressors and a random intercept. Correction for multiple comparisons was performed using FDR correction across 50 subnuclei/tracts. Correlational analyses of subnucleus volumes or mean tract FC with UM-PDHQ scores (indicating hallucination severity) was performed using Spearman correlation coefficient.

To ensure that anxiety and depression did not drive the effect seen in PD-VH, we also performed correlation analyses (Spearman correlation coefficient) of subnucleus volumes and tract FC with Hospital Anxiety and Depression Scale (HADS) scores. Statistical analyses were performed in Python V.3 using Jupyter Lab V.1.2.6.

## Results

A total of 101 participants were included; 76 patients with PD, of whom 15 PD-VH and 61 PD non-VH, and 26 controls. Demographics and results of clinical assessments at baseline are shown in table 1. The groups were well matched in age, gender, years in education. PD-VH and PD non-VH did not differ in terms of baseline cognitive performance, except lower scores in Stroop (both colour and interference) in PD-VH. PD-VH had higher prevalence of other non-motor symptoms with higher anxiety and depression scores and higher total UPDRS scores (U=289, p=0.014) but did not differ in motor severity, disease duration or levodopa-equivalent daily dose (table 1). Time interval between the two scans (baseline and visit 2) was 1.17 to 1.67 years (mean 1.28, SD 0.08) with no significant between-group differences.

**Table 1:** Demographics and results of clinical assessments at baseline

Characteristic	Controls n=26	PD non-VH n=61	PD-VH n=15	P value
Age (years)	67.4 (8.2)	64.6 (8.1)	64.5 (8.0)	0.308
Male (%)	12 (46.2)	36 (59.0)	6 (40%)	0.302
Years of education	17.9 (2.2)	17.3 (2.6)	16.9 (3.8)	0.686
Vision				
Contrast sensitivity (Pelli Robson)§	1.8 (0.2)	1.8 (0.2)	1.7 (0.2)	0.136
Acuity (LogMar)§	−0.08 (0.2)	−0.07 (0.2)	−0.08 (0.1)	0.136
Colour vision (D15)§	1.2 (1.0)	1.2 (1.0)	1.6 (1.8)	0.927
General cognition				
MOCA	29 (1.2)	28.0 (2.3)	27.6 (1.8)	0.050‡
MMSE	29.2 (0.9)	29.0 (1.2)	28.9 (1.3)	0.847
Mood				
HADS anxiety	**3.5** (**3.5**)	**5.1** (**3.5**)	**8.0** (**4.2**)	0.002*†
HADS depression	**1.2** (**1.5**)	**3.7** (**3.1**)	**4.8** (**3.3**)	**<0.001†‡**
Detailed neuropsychology				
Attention				
Digit span forwards	9.3 (2.1)	9.1 (2.0)	10 (2.0)	0.412
Digit span backwards	6.9 (2.4)	7.3 (2.2)	7.6 (2.4)	0.748
Stroop: colour (sec)	**31.9** (**7.6**)	**32.6** (**6.4**)	**38.3** (**8.5**)	0.012*‡
Executive function				
Stroop: interference (sec)	**56.2** (**14.3**)	**40.3** (**20.1**)	**72.9** (**26.6**)	0.029*‡
Category fluency	21.9 (4.8)	22.1 (6.0)	20.1 (4.0)	0.502
Memory				
Word recognition task	24.5 (1.0)	24.3 (2.3)	23.8 (1.2)	0.077
Logical memory (delayed)	12.8 (3.5)	13.3 (4.6)	13.5 (4.5)	0.928
Language				
Graded naming task	23.6 (1.0)	24.3 (2.5)	23.5 (3.1)	0.638
Letter fluency	17.8 (5.1)	17.3 (5.3)	16.1 (4.9)	0.711
Visuospatial				
Benton’s judgement of line orientation	26 (3.4)	25.3 (3.7)	23.1 (4.8)	0.134
Hooper	25.9 (2.1)	25.0 (2.9)	23.9 (3.1)	0.082
Disease-specific measures				
Years from diagnosis	–	4.0 (2.5)	4.5 (2.7)	0.238
UPDRS total score	–	**42.9** (**19.4**)	**57.8** (**24.3**)	**0.014**
UPDRS motor score	–	22.7 (11.7)	26.2 (15.2)	0.052
Right side affected at onset	–	28 (45.9)	4 (26.7)	0.187
RBDSQ	–	**3.8** (**2.1**)	**5.7** (**2.4**)	**0.003**
Sniffin sticks	–	**7.6** (**2.9**)	**6.9** (**3.4**)	**0.003**
LEDD	–	427.1 (220.1)	431.0 (233.1)	0.951

All data shown are mean (SD) except gender and affected size.

In bold characteristics that significantly differed between groups.

For all neuropsychology measures, higher scores indicate better performance, except Stroop Colour and Interference where lower scores imply better performance.

*Statistically significant difference between PD-VH and PD non-VH.

†Statistically significant difference between PD non-VH and controls.

‡Statistically significant difference between PD-VH and controls.

§Best binocular score used; LogMAR and D15: lower score implies better performance, Pelli-Robson: higher score implies better performance.

HADS, Hospital Anxiety and Depression Scale (higher scores indicate increased anxiety and depression) LEDD, levodopa equivalent dose; MMSE, Mini-Mental State Examination; MOCA, Montreal Cognitive Assessment; PD, Parkinson’s disease; RBDSQ, REM sleep behaviour disorder scale; UPDRS, Unified Parkinson’s Disease Rating Scale; VH, visual hallucination.

During follow-up, PD-VH showed greater worsening performance than PD non-VH in measures of general cognition (MOCA t=2.930, p=0.005; MMSE Mann-Whitney U=317.5, p=0.030). Table 2 shows the longitudinal changes in general cognition and motor symptoms in PD participants (for details on longitudinal cognitive performance, see online supplemental table 1).

**Table 2:** Longitudinal changes in patients with Parkinson’s disease without hallucinations (PD non-VH) and patients with hallucinations (PD-VH)

Cognitive test	PD non-VH n=61	PD-VH n=15	PD non-VH n=61	PD-VH n=15	Statistic
General cognition	Baseline visit		Follow-up visit (18 months)		P value*
MOCA	**28.0** (**2.3**)	**27.6** (**1.8**)	**28.1** (**2.1**)	**25.5** (**5.2**)	**t=2.93** **p=0.005**
MMSE	**29.0** (**1.2**)	**28.9** (**1.3**)	**29.1** (**1.0**)	**27.7** (**3.2**)	**U=317.5** **p=0.030**
Motor symptoms					
UPDRS total score	42.9 (19.4)	57.8 (24.3)	41.8 (6.3)	58.4 (17.2)	t=0.345 p=0.731
UPDRS motor score	22.7 (11.7)	26.2 (15.2)	21.7 (10.2)	26.1 (10.2)	t=0.244 p=0.808
LEDD	427.1 (220.1)	431.0 (233.1)	427.1 (220.1)	431.0 (233.1)	–
Hallucinations					
Weekly visual hallucinations	–	14 (93.3)	–	12 (80.0)	x^2^=0.001 p=0.985
UM-PDHQ	–	3.5 (2.9)	–	3.7 (3.2)	t=0.179 p=0.859

All data shown are mean (SD) except the presence of weekly visual hallucinations which is presented as n (%).

No difference was seen in any individual cognitive tests longitudinally between PD-VH and PD non-VH; results of individual cognitive tests per domain are presented in online supplemental table 2).

UM-PDHQ: University of Miami Parkinson’s disease Hallucinations Questionnaire: higher scores indicate more severe hallucinations.

*Statistical comparison of individual performance change (performance in follow-up visit – performance in baseline visit) for each metric; using t-test for normally distributed variables and Mann-Whitney for non-normally distributed variables. In bold characteristics that significantly differed in terms of change between visit 2 and baseline between groups.

LEDD, levodopa dose equivalence score; MMSE, Mini-Mental State Examination; MOCA, Montreal Cognitive Assessment; PD, Parkinson’s disease; UPDRS, Unified Parkinson’s Disease Rating Scale; VH, visual hallucination.

### Cortical grey matter is relatively preserved in PD with VH despite widespread white matter macrostructural changes

**Figure 1:**
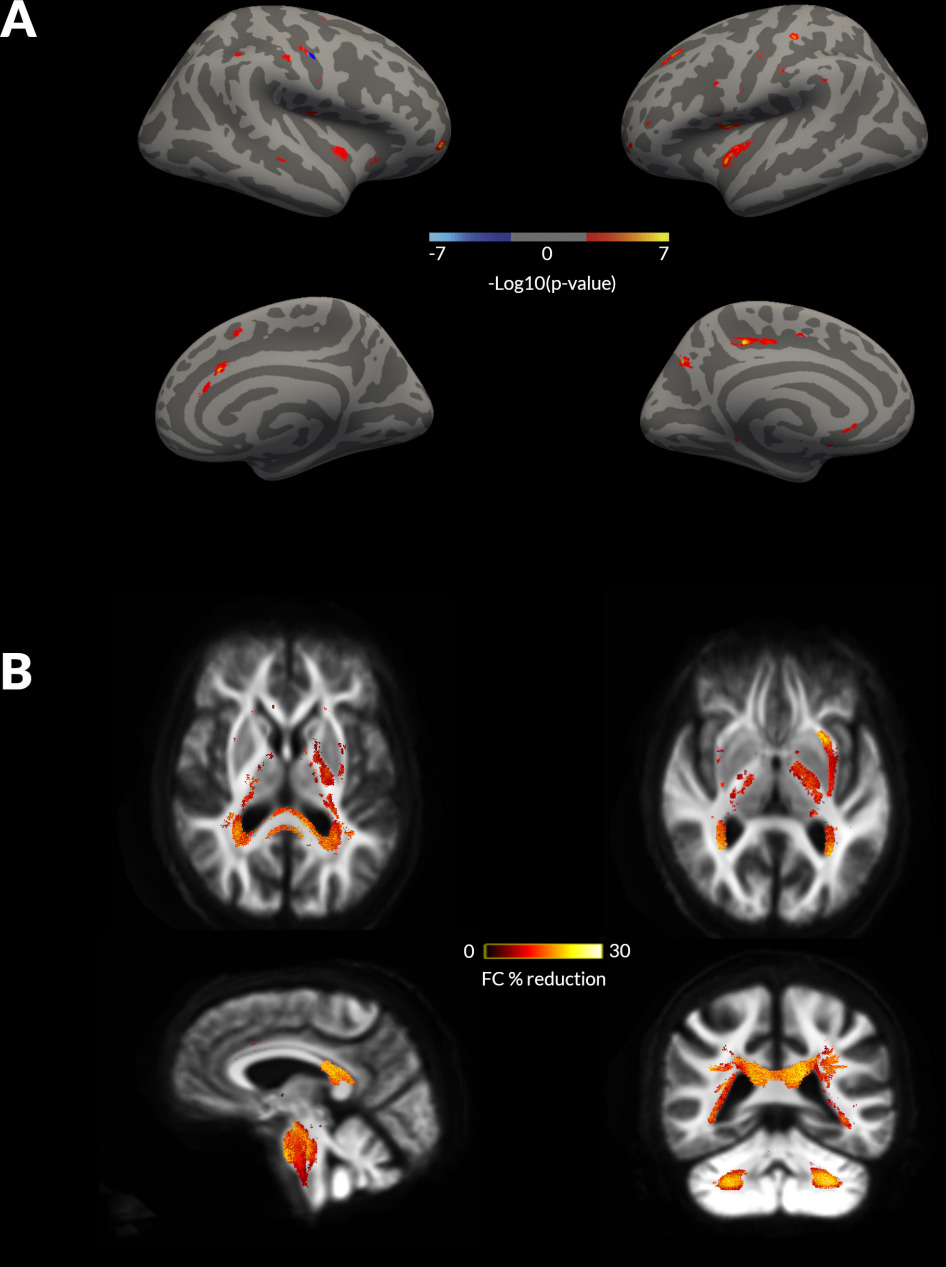
Whole brain, grey matter and white matter changes in patients with Parkinson’s disease (PD) and visual hallucinations (VH) at 18 months follow-up. (A) Changes in cortical thickness seen in patients with compared with those without hallucinations (PD non-VH) at 18 months follow-up, on a surface rendered right hemisphere (left: lateral view top panel, medial view bottom panel) and left hemisphere. No statistically significant changes were seen in cortical thickness at baseline imaging. Colour coding indicates cluster significance for group-by-time interactions. Significance levels are on a logarithmic scale of p values (−log10). Positive values indicate PD-VH cortical thickness <PD non-VH; negative values indicate PD-VH >PD non VH. Results are corrected for false discovery rate across both hemispheres. (B) Changes in white matter macrostructure (fibre cross-section, FC) seen in PD-VH compared with PD non-VH at longitudinal follow-up. Baseline changes are presented in online supplemental figure 1. Results are displayed as streamlines; these correspond to fixels that significantly differed between PD low and high visual performers (family-wise error correction (FWE)-corrected p<0.05). Streamlines are coloured by percentage reduction (colourbars) in PD-VH compared with PD non-VH.

At baseline, no statistically significant differences in cortical thickness were seen between PD with and without hallucinations. Cortical thickness for both PD-VH and PD non-VH decreased over 18 months follow-up, with significantly higher reductions longitudinally in PD-VH compared with PD non-VH with clusters including the left precuneus, bilateral anterior cingulate, bilateral precentral and postcentral gyrus, bilateral superior frontal and anterior cingulate gyrus, bilateral insula, right supramarginal gyrus, right superior temporal gyrus and right lateral occipital gyrus (figure 1A).

For white macro- and micro-structure, PD-VH also showed significant changes compared with PD without hallucinations, longitudinally (figure 1B), and some changes were already present at baseline, as we have previously shown.^10^ Specifically, at baseline, PD-VH showed macrostructural changes (FC reductions) and microstructural changes (FD reductions) within the splenium of the corpus callosum and the left posterior thalamic radiation. Reductions were also seen in the combined FDC metric across the same regions, particularly within the splenium which showed over 30% FDC reduction in PD-VH compared with PD non-VH (online supplemental figure 1).

Longitudinally, there were additional extensive macrostructural changes (FC reductions) in PD-VH compared with PD non-VH within the splenium, bilateral posterior thalamic radiations, bilateral posterior internal capsules, bilateral tapetum, left inferior fronto-occipital fasciculus, and left superior longitudinal fasciculus (figure 1B). No differences in the longitudinal reduction of FD or FDC were seen between groups.

### Specific volume loss of the right mediodorsal medial thalamic nucleus is seen in PD-VH longitudinally and is preceded by respective white matter connection loss

**Figure 2:**
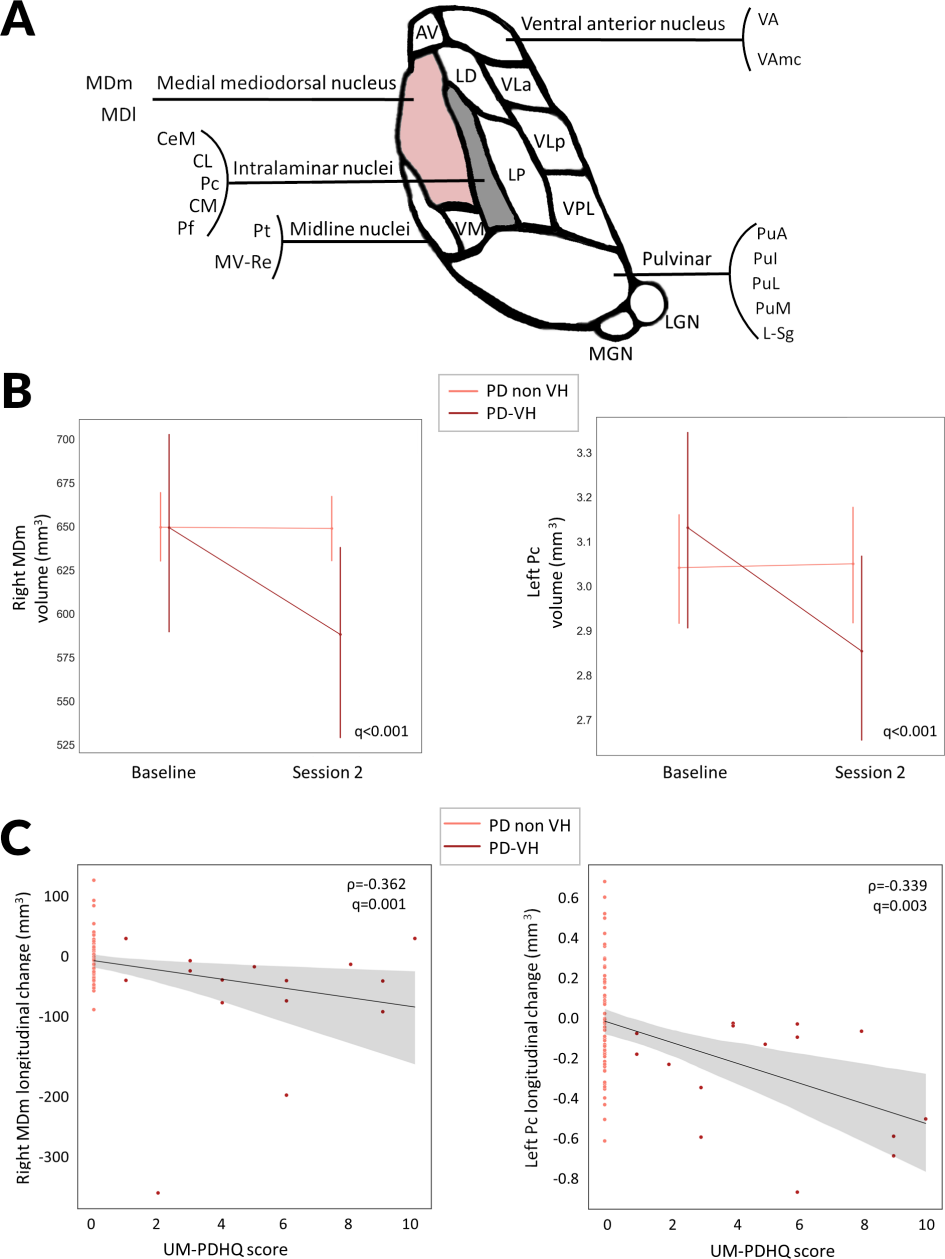
Thalamic subnucleus specific changes in PD with visual hallucinations compared with PD without hallucinations at 18 months follow-up. (A) Diagrammatic representation of the human thalamic subnuclei. Highlighted in pink are the nuclei showing significant longitudinal changes in volume in PD with visual hallucinations (PD-VH) compared with PD without hallucinations (PD non-VH). The medial mediodorsal nucleus is further subdivided into mediodorsal medial parvocellular and mediodorsal medial magnocellular nuclei. Intralaminar nuclei include the Pc, Pf, CeM, CL and CM. (B) Longitudinal change in thalamic nuclei volumes for the right MDm and the left Pc in PD-VH, PD nonVH in mm^3^. Corrected for age, gender, total intracranial volume and time between scan. Family wise error (FDR) corrected p-value presented for the group-by-time interaction comparison between PD-VH and PD non-VH participants. Error bars represent SD. (C) Change in thalamic nuclei volumes for the right MDm and the left Pc in PD participants was correlated with severity of visual hallucinations, assessed using the University of Miami Parkinson’s disease Hallucinations Questionnaire. Higher scores indicating more severe hallucinations. AV, anteroventral; CeM, central medial; Cl, central lateral; CM, centromedian; L_Sg, limitans; LD, laterodorsal; LGN, lateral geniculate; LP, lateral posterior; MDl, mediodorsal medial parvocellular; MDm, mediodorsal medial magnocellular; MGN, medial geniculate; MVRe, reuniens medial ventral; PD, Parkinson’s disease; Pf, parafascicular; PuA, pulvinar anterior; PuI, pulvinar inferior; PuL, pulvinar lateral; PuM, pulvinar medial; VA, ventral anterior; VAmc, ventral anterior magnocellular; VLa, ventral lateral anterior; VLp, ventral lateral posterior; VPL, ventral posterolateral; VM, ventromedial; ρ, Spearman correlation coefficient.

Thalamic volumes (either whole thalamic or subnucleus volume) at baseline showed no significant difference between PD-VH and PD non-VH, correcting for age, gender and total intracranial volume, after correction for multiple comparisons. However, when assessing differences in longitudinal thalamic subnuclei volumes, PD-VH showed significantly higher reductions in volume of the right medial mediodorsal magnocellular nucleus (MDm: t=−3.018, FDR-corrected p value, q<0.001) and the left Pc nucleus (Pc: t=−3.490, q<0.001) compared with PD non-VH (figure 2, table 3). Thalamic subnucleus volume loss was significantly correlated with hallucination severity (mean UM-PDHQ score across both visits) for both the right MDm (Ρ=−0.362, p=0.001) and the left Pc nucleus (Ρ=−0.339, p=0.003).

**Table 3:** Grey matter areas showing significant longitudinal differences in cortical thickness or thalamic nucleus volumes between PD patients with visual hallucinations (PD-VH) and those without hallucinations (PD non-VH)

Changes in cortical thickness							
Tailarach coordinates			Hemisphere	Anatomical location	Number of vertices	Size (mm^2^)	Zmax
x	y	z					
28.1	−20.8	37.8	L	Precuneus	368	140.54	7.5
−32.7	55.4	14.2	R	Caudal anterior cingulate	136	56.87	7.4
29	13.8	27.1	R	Precentral	143	39.47	7.4
3.5	92.5	−31	R	Rostral middle frontal	115	57.25	7.3
27.7	−66.5	28.8	L	Superior frontal	134	55.16	7.2
−16	29.5	−12.3	L	Insula	184	45.62	7.1
−20.4	−11	42.5	L	Postcentral	113	29.17	7.1
−15.8	28.4	−31.5	L	Insula	343	136.58	7
5.7	67.6	30.9	L	Superior frontal	196	98.74	6.9
22.8	12.3	−5.5	R	Postcentral	126	31.91	6.7
−30.2	42.5	41.1	R	Superior frontal	53	27.29	6.7
38.1	48.9	−28.4	L	Rostral anterior cingulate	50	29.98	6.6
16.7	25.3	−27.5	R	Insula	152	57.93	6.6
28.3	−29.1	31.3	R	Supramarginal	91	24.73	6.6
35.8	0.5	25.4	R	Postcentral	84	22.18	6.4
3.5	−86.9	−28.8	R	Lateral occipital	19	9.09	6.3
30.2	24.7	38.3	L	Superior frontal	50	15.03	6.1
−33.5	62.6	4.4	R	Caudal anterior cingulate	50	29.73	6.1
39.4	−7	−29.4	R	Superior temporal	29	9.06	6.1
−4	93.4	−27.3	L	Rostral middle frontal	41	21.08	6
−27.7	36.1	11.5	L	Precentral	25	9.54	5.9

Changes in thalamic subnucleus volume				
Thalamic subnucleus	Hemisphere	Beta	P value	Q value
Mediodorsal medial magnocellular	R	−0.037	0.002	0.05
Paracentral	L	−0.016	9.85E-06	<0.001

Anatomical locations extracted from aparc freesurfer annotation.

Zmax indicates the −log10 (p value) for the cluster, a threshold of 5.613 was calculated to represent false discover rate (FDR) corrected values p<0.05 for both hemispheres.

No statistically changes were seen at baseline imaging between PD-VH and PD non-VH participants.

**Figure 3:**
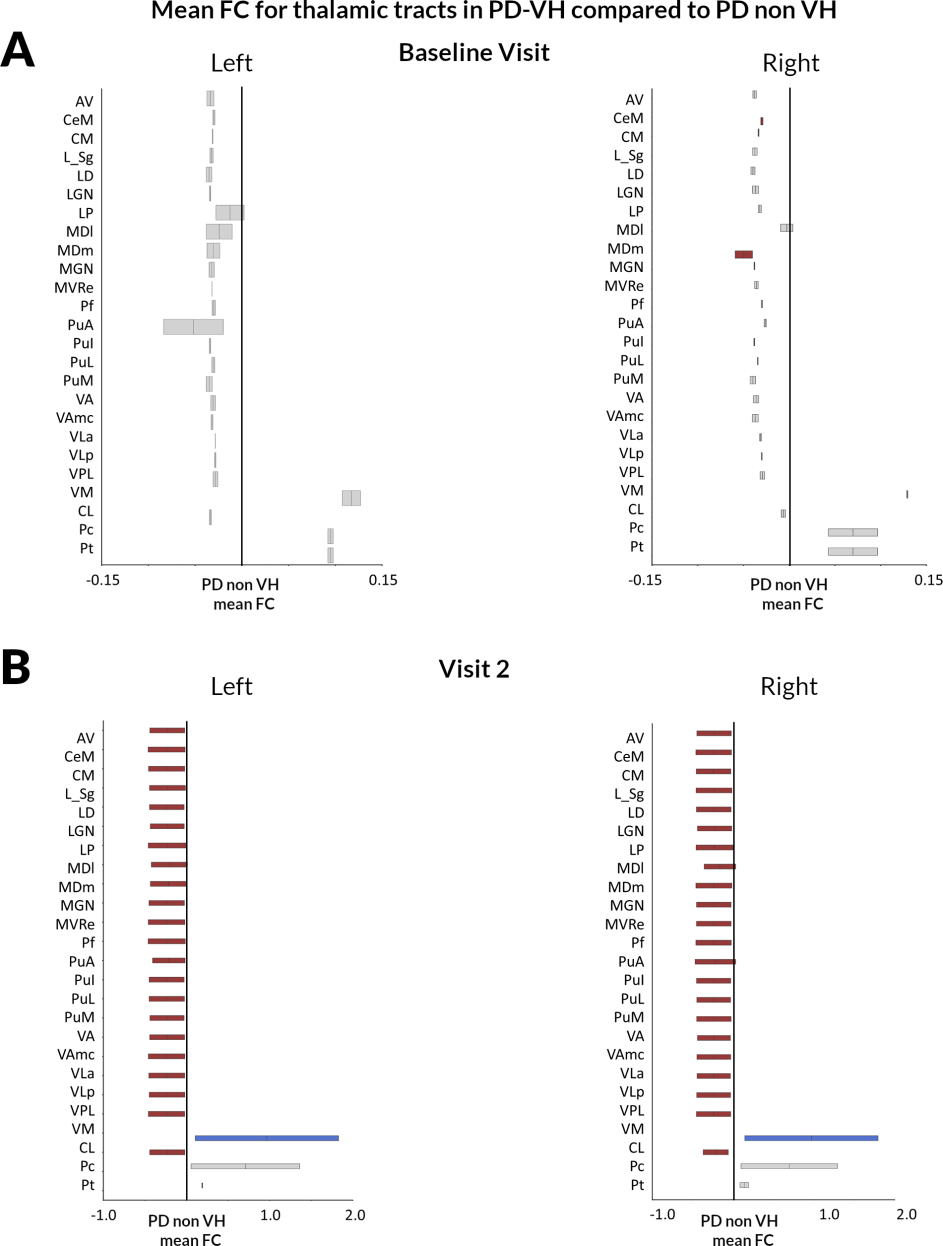
Thalamic tract-of-interest changes in FC in PD-VH compared with those without hallucinations at baseline and during longitudinal follow-up. (A) Baseline visit. Reduction (mean, 95% CI) in FC visualised for patients with PD-VH compared with patients with PD without hallucinations (PD non-VH). Tracts with significantly reduced FC (FDR-corrected p value<0.05) are shown in dark red (right MDm and right CeM), while tracts where there are no significant changes in FDC are plotted in grey. (B) Visit 2 (18 months follow-up). Reduction (mean, 95% CI) in FC visualised for PD-VH compared with PD non-VH. Tracts with significantly reduced FC (FDR-corrected p value<0.05) are shown in colour, while tracts with no significant changes in FDC are plotted in grey. White matter tracts from all but three thalamic subnuclei (Pc, Pt and VM bilaterally) showed more severe volume loss in PD-VH compared with PD non-VH at longitudinal follow-up. Bilaterally tracts originating in the VM nuclei showed significantly less volume loss in PD-VH compared with PD non-VH. AV, anteroventral; CeM, central medial; CL, central lateral; CM, centromedian; L_Sg, limitans; LD, laterodorsal; LGN, lateral geniculate; LP, lateral posterior; MDl, mediodorsal medial parvocellular, MDm, mediodorsal medial magnocellular; MGN, medial geniculate; MVRe, reuniens medial ventral; Pc, paracentral; PD, Parkinson’s disease; Pf, parafascicular; PuA, pulvinar anterior; PuI, pulvinar inferior; PuL, pulvinar lateral; PuM, pulvinar medial; VA, ventral anterior; VAmc, ventral anterior Pt, paratenial; magnocellular; VH, visual hallucination; VLa, ventral lateral anterior; VLp, ventral lateral posterior; VPL, ventral posterolateral; VM, ventromedial.

L, left; PD, Parkinson’s disease; R, right.VH, visual hallucination;

White matter tracts-of-interest from thalamic subnuclei to the ipsilateral cortex showed significant reductions in mean FC in PD-VH compared with PD non-VH already at baseline; specifically in the right mediodorsal medial magnocellular nucleus (MDm: t=−0.037, FDR-corrected q=0.05) and the right centromedial nucleus (CeM: t=−0.0157, q<0.001) (figure 3A). Longitudinally, tracts-of-interest from 44 out of 50 thalamic subnuclei showed significant reductions in mean FC in PD-VH compared with PD non-VH, adjusting for age, gender, total intracranial volume and time between scans and FDR corrected across 50 tracts (figure 3B). The Pc and paratenial nuclei bilaterally did not show significant differences in PD-VH longitudinally (FDR-corrected q>0.05) and bilaterally the ventromedial nuclei showed significantly less volume loss in PD-VH (right VM: t=2.964, q=0.006, left VM: t=2.952, q=0.006). All other thalamic tracts-of-interest (88%) showed greater volume loss in PD-VH compared with PD non-VH; longitudinal reduction in mean FC was significantly correlated with hallucination severity (Ρ=−0.212, q<0.001). Details on the longitudinal changes in thalamic tract mean FC are seen in online supplemental table 2.

Anxiety and depression scores (using the HADS) were not correlated with MDm subnucleus volume or mean tract FC at baseline or longitudinally (see online supplemental table 3 and online supplemental figures 2 and 3).

## Discussion

This study sheds light on the timeline and spatial profile of structural thalamic involvement in patients with PD and VH. We have shown that: (a) the right mediodorsal medial thalamus is affected in PD with hallucinations, with white matter tracts connected to the right mediodorsal thalamus showing macrostructural changes (reduced FC) at baseline and volume loss within the nucleus after 18 months, (b) widespread white matter macrostructural changes develop over time involving the majority of thalamocortical white matter tracts and (c) white matter changes associated with PD-hallucinations precede loss of cortical thickness, with whole-brain white matter changes already seen at baseline but differences in cortical thickness only evolving after follow-up.

By using a recently described probabilistic atlas derived from ex vivo imaging and histology,^20^ we were able to detect differences in volumes within the right mediodorsal medial thalamic subnucleus in PD with hallucinations. The mediodorsal medial nucleus is a higher order, associative thalamic nucleus with multiple reciprocal connections with the prefrontal and anterior cingulate cortex.^42^ Its role in cognition is now well established, particularly in sustaining PFC activity during working and spatial memory^43^ and monitoring and updating mental representations.^13^ It has also been implicated in psychiatric disease; patients with schizophrenia show reduced functional activation and associated reduced functional connectivity to the PFC during executive tasks;^44^ they also show grey matter atrophy in the mediodorsal thalamus.^45 46^ Reduced functional connectivity of the mediodorsal thalamus with the paracingulate and posterior cingulate has also been described in patients with Parkinson’s and cognitive impairment.^47^ The complex way that the mediodorsal medial nucleus interacts with the PFC is not fully understood, however, there is evidence to suggest that it may act as a regulator of PFC function:^48^ reduced input from the mediodorsal medial nucleus, due to white matter degeneration and neuronal loss within the nucleus could result in subsequent unregulated PFC activity.

In whole-brain fixel-based analysis, we saw a significant posterior predominance of white matter changes in PD-VH: changes in the splenium of the corpus callosum and posterior thalamic radiations seen at baseline progressed during follow-up to involve multiple tracts such as the tapetum and posterior internal capsules but frontal connections remained relatively preserved. Reduced connectivity between subcortical regions and visuospatial regions when combined with unregulated PFC activity (due to reduced control from the mediodorsal medial thalamic nucleus), which retains its other cortical white matter projections, may partly explain the overweighting of prior knowledge seen in PD-hallucinators.^8^

In addition, significant longitudinal changes were seen in infratentorial regions, particularly in bilateral middle cerebellar peduncles. Increasing evidence has demonstrated a potential crucial role for the cerebellum in the development of hallucinations: atrophy of the cerebellum has been described in patients with PD and VH,^49–51^ reduced metabolism of the vermis has been shown in patients with hallucinations secondary to Lewy body disorders,^52^ and a recent study of lesional hallucinations using network lesion mapping revealed a common network associated with hallucinations, with connectivity to the cerebellar vermis and inferior cerebellum.^53^ The cerebellum plays an important role in cognition, crucially by updating predictive models of behaviours through error learning^54^ with sensitivity to errors associated with the activity of the cerebellar vermis.^55^ Loss of grey and white matter within the cerebellum may lead to a reduced sensitivity to prediction errors and may contribute to the relative overweighting of prior knowledge seen during visual perception in PD hallucinators.^8^

Interestingly, both in whole-brain and thalamic analyses, we saw white matter macrostructural changes in PD-VH before any changes in cortical thickness or thalamic volume loss were evident: patients with PD-VH showed significant macrostructural (reduced FC) and microstructural changes (reduced FD) within posterior white matter tracts already at baseline, in the absence of any cortical thickness changes. In addition, the right mediodorsal medial thalamus, which showed reduced volume in PD-VH at follow-up, showed macrostructural changes in its connections with the cortex at baseline.

Although, this result could be due to different sensitivities of the imaging modalities used to assess grey and white matter, it provides further support for the important role white matter degeneration plays in PD. Axonal pathology has been demonstrated prior to dopaminergic neuronal loss in animal^22 56^ and cell models.^57^ Alpha-synuclein plays a role in axonal growth with higher density, thinner axons seen in the brain of patients with early PD.^58^ Imaging biomarkers that assess white matter integrity such as fixel-based analysis might be more sensitive at picking up anatomical abnormalities at the earliest stages of PD; our findings support this.

Our finding of changes in thalamic grey and white matter in PD with hallucinations could underlie the more widespread network differences found in PD hallucinators.^6 11 12^ The medial mediodorsal nucleus, which showed volume reduction in PD hallucinators, is a feasible target for deep brain stimulation, which has been performed in small numbers of patients with severe obsessive compulsive disorder.^59^ Given the changes in white matter connectivity from the medial mediodorsal nucleus to the cortex seen in PD hallucinators, further work in the connectivity between this subnucleus and PFC, in particular, could yield possible connectomic targets for deep brain stimulation^60^ to treat hallucinations in PD.

Although the LGN has previously been functionally implicated in PD hallucinations,^53 61^ we did not find specific volume or tract reductions for the LGN at baseline, although reductions in tracts connected to the LGN were seen longitudinally. It is possible that changes in functional connections with the LGN do not affect structural integrity until later stages.

Several methodological considerations need to be considered when interpreting our findings. Our participants underwent imaging acquisition while continuing their usual dopaminergic medications. Given we are assessing structural metrics, it is unlikely that these will be affected by medication and levodopa equivalent doses did not differ between PD-VH and PD non-VH. The number of hallucinators in our cohort is consistent with reports from other groups showing that minor hallucinations can be seen even in patients recently diagnosed with PD.^62^ Due to the imaging acquisition protocols in our study, we could not formally quantify the presence of white matter hyperintensities. Although no studies using fixel-based analysis have specifically controlled for white matter hyperintensities,^10 38 40^ if present, these are likely to decrease FD.^63^ It is not clear if white matter hyperintensities could have an effect on FC, which was our primary metric to assess white matter longitudinally; this could be clarified in future studies. Hallucinations in PD are associated with other non-motor symptoms and worsening cognition;^1 2 5^ similarly, in our cohort, PD-VH had higher rates of anxiety and depression. Although HADS scores were not correlated with MDm volume or tract FC at baseline or longitudinally, the structural changes identified in our study could be influenced by non-motor symptoms other than hallucinations.

Patients with PD and VH show both white matter and grey matter degeneration longitudinally with changes in metrics of white matter macrostructure such as fibre cross-section occurring before loss of cortical thickness. In addition, we show that thalamic cortical connectivity is affected in Parkinson’s-associated hallucinations, particularly within the mediodorsal nucleus. Our findings provide mechanistic support for the role of the thalamus as a driver of network imbalance in Parkinson’s hallucinations and support the use of imaging techniques aimed at white rather than grey matter in assessing early stages of PD.

## Data Availability

Data are available in a public, open access repository. Data are available upon reasonable request. All data relevant to the study are included in the article or uploaded as supplementary information. All code used to perform the analyses presented in this study is shared freely as a github repository: https://github.com/AngelikaZa/ThalamicSubnuclei. Anonymized data will be shared on request, for purposes of replicating procedures and results.
