# Supplementary material for: Longitudinal thalamic white and grey matter changes associated with visual hallucinations in Parkinson’s disease
Source: J Neurol Neurosurg Psychiatry. 2021 Sep 28;93(2):169–79. doi: 10.1136/jnnp-2021-326630 (PMC8785065; doi:10.1136/jnnp-2021-326630)
Supplement: Supplementary data [file jnnp-2021-326630supp001.pdf]

Supplementary Material: Longitudinal thalamic white and gray matter changes associated with visual hallucinations in Parkinson's disease

Angeliki Zarkali MBBS[1], Dr Peter McColgan PhD[2], Louise-Ann Leyland PhD[1], Andrew J. Lees F.Med.Sci [3], Rimona S. Weil PhD[1,4,5]

Contents

Supplementary Methods 2
Supplementary Results 3
Supplementary Figure 1. 3
Supplementary Table 1. 4
Supplementary Table 2. 6
Supplementary Table 3. 8
Supplementary Figure 2. 9
Supplementary Figure 3. 10
References 11

Supplementary Methods

Excluded participants

Participants were recruited to the National Hospital, Queen Square and underwent clinical assessments and brain imaging at baseline and after 18 months (Visit 2). Only participants who had structural and diffusion-weighted imaging satisfying pre-determined quality control criteria at both visits were included. All volumes of raw datasets for both structural T1-weighted imaging and diffusion weighted imaging were visually inspected and evaluated for the presence of artefact; only scans with <15 volumes containing artefacts[1] were included.

A total of 140 participants were imaged at baseline. At Visit 2, 4 patients withdrew from the study, 11 participants were unable to be scanned due to evolving contra-indications or participant request, 3 were excluded due to a subsequent revised diagnosis of atypical parkinsonian syndrome, 19 were unable to participate due to COVID-19 and 2 failed quality control for Visit 2 diffusion-weighted imaging. Therefore 101 participants were included in the study: 76 patients with PD and 25 controls (from spouses and volunteer databases). All patients with PD, satisfied the Queen Square Brain Bank criteria[2].

Neuropsychometry and clinical assessments

All participants underwent assessments of general cognition using the Mini-Mental State Examination (MMSE) and Montreal Cognitive Assessment (MoCA)[3,4]. Domain specific cognitive assessments were also performed using two tests per cognitive domain. Attention: Digit span backwards[5] and Stroop: Naming[6], Executive function: Stroop Interference[6] and Category fluency[7], Memory: Word Recognition Task[8] and Logical Memory[5], Language: Graded Naming Task[9] and Letter fluency[7] and Visuospatial: Benton's Judgment of Line[10] and Hooper Visual Organization Test[11]. Visual acuity was assessed using LogMAR[12], colour vision using the D15[13] and contrast sensitivity using the Pelli-Robson test[14]. Motor function was assessed using the MDS-UPDRS[15]. Smell was assessed using Sniffin' Sticks[16]. Mood was assessed using the Hospital Anxiety and Depression Scale (HADS)[17] and sleep using the REM Sleep Behaviour Disorder Questionnaire (RBDSQ)[18]. Levodopa dose equivalence scores (LEDD) were calculated for PD participants[19].

Supplementary Results

Supplementary Figure 1.

Fibre tract-specific reductions at baseline in PD with hallucinations (PD-VH) compared to PD without hallucinations (PD non VH).

PD-VH showed macrostructural changes (changes in fibre cross-section (FC)) within the splenium of the corpus callosum and bilateral posterior thalamic radiations. Microstructural changes (reductions in fibre density (FD)) were also seen at baseline imaging, with FD reductions in PD-VH in the splenium of the corpus callosum, bilateral posterior thalamic radiations and the right corticospinal tract. Changes in the combined FDC metric were seen within the body and splenium of the corpus callosum, posterior thalamic radiations bilaterally, and the right corticospinal tract; these represent impaired overall ability to relay information in these tracts in PD hallucinators.

Results are displayed as streamlines (FWE-corrected p <0.05). Streamlines are coloured by percentage reduction (colourbars) in PD-VH compared to PD non VH.

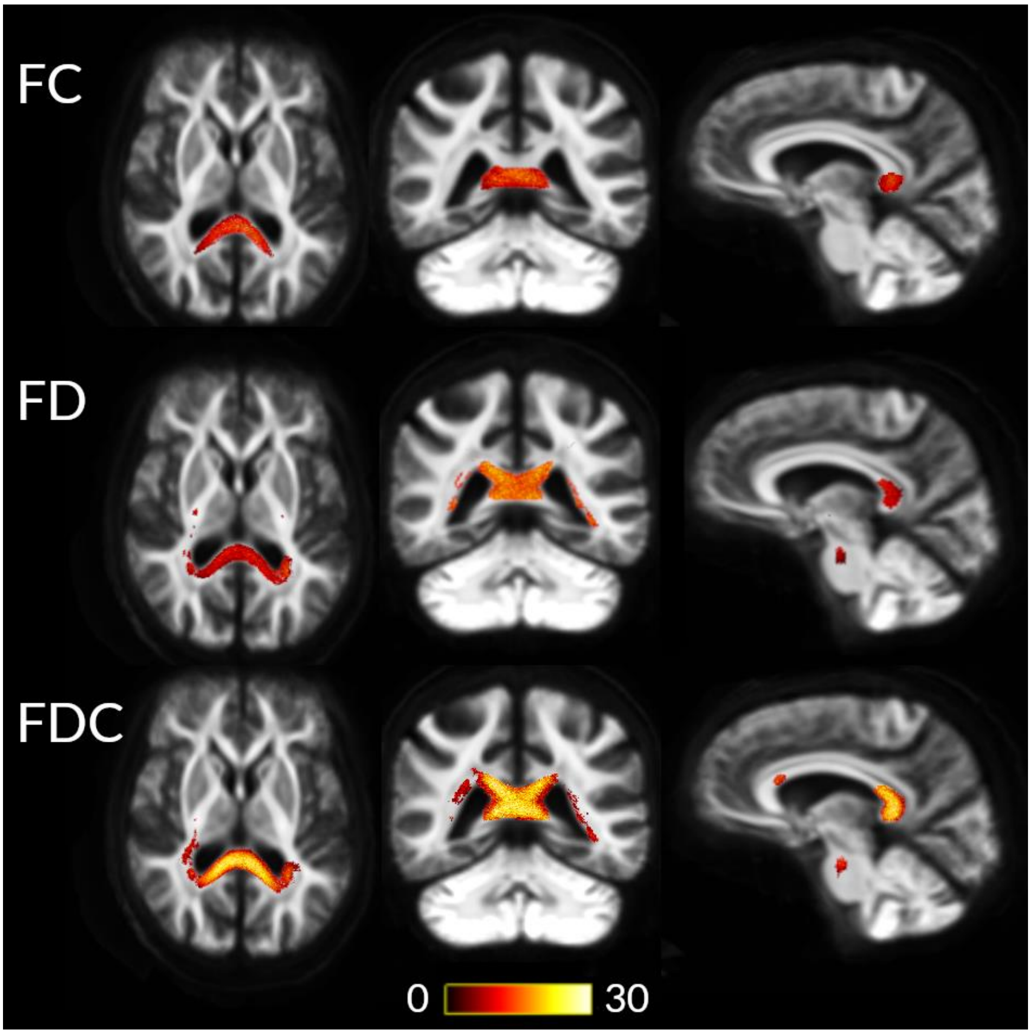

Supplementary Table 1.

Longitudinal changes in cognition and motor severity between patients with Parkinson's disease without hallucinations (PD non VH) and patients with hallucinations (PD VH).

Cognitive test	PD non VH n= 61	PD VH n= 15	PD non VH n= 61	PD VH n= 15	Statistic
General cognition	**Baseline visit**		**Follow up visit (18 months)**		**p-value***
MOCA	**28.0 (2.3)**	**27.6 (1.8)**	**28.1 (2.1)**	**25.5 (5.2)**	**t=2.93 p=0.005**
MMSE	**29.0 (1.2)**	**28.9 (1.3)**	**29.1 (1.0)**	**27.7 (3.2)**	**U=317.5 p=0.030**
Attention					
Digit span forwards	9.1 (2.0)	10 (2.0)	9.9 (1.7)	9.3 (2.1)	U=181 p=0.051
Digit span backwards	7.3 (2.2)	7.6 (2.4)	7.9 (2.3)	7.6 (2.6)	t=0.923 p=0.359
Stroop: Colour (sec)	32.6 (6.4)	38.3 (8.5)	33.6 (8.7)	40.2 (3.8)	U=394 p=0.362
Executive function					
Stroop: Interference (sec)	40.3 (20.1)	72.9 (26.6)	59.1 (22.5)	70.7 (19.7)	U=373 p=0.406
Category fluency	22.1 (6.0)	20.1 (4.0)	20.4 (5.5)	18.8 (7.1)	t=0.269 p=0.789
Memory					
Word Recognition Task	24.3 (2.3)	23.8 (1.2)	24.1 (1.4)	24.0 (1.0)	U=347.5 p=0.131
Logical Memory (delayed)	13.3 (4.6)	13.5 (4.5)	12.7 (3.6)	13.0 (4.6)	t=0.293 p=0.770
Language					
Graded Naming Task	24.3 (2.5)	23.5 (3.1)	24.8 (2.9)	23.3 (4.1)	U=437 p=0.395
Letter Fluency	17.3 (5.3)	16.1 (4.9)	16.7 (4.4)	16.2 (6.1)	t=-0.590 p=0.557
Visuospatial					
JLO	25.3 (3.7)	23.1 (4.8)	25.3 (3.7)	21.9 (4.8)	U=265 p=0.066
Hooper	25.0 (2.9)	23.9 (3.1)	25.2 (3.2)	23.2 (5.0)	U=424 p=0.333
Motor Symptoms					
UPDRS total score	42.9 (19.4)	57.8 (24.3)	41.8 (6.3)	58.4 (17.2)	t=0.345 p=0.731
UPDRS motor score	22.7 (11.7)	26.2 (15.2)	21.7 (10.2)	26.1 (10.2)	t=0.244 p=0.808
LEDD	427.1 (220.1)	431.0 (233.1)	427.1 (220.1)	431.0 (233.1)	-

Hallucinations					
Weekly visual hallucinations	-	14 (93.3)	-	12 (80.0)	$x^2 = 0.001$ p=0.985
UM-PDHQ	-	3.5 (2.9)	-	3.7 (3.2)	t=0.179 p=0.859

All data shown are mean (SD) except the presence of weekly visual hallucinations which is presented as n (%).
** Statistical comparison of individual performance change (Performance in Follow up visit – Performance in Baseline visit) for each metric; using t test for normally distributed variables and Mann-Whitney for non-normally distributed variables. In bold characteristics that significantly differed in terms of change between Visit 2 and baseline between groups.*
JLO: Judgement of Line Orientation. For all neuropsychology measures higher scores indicate better performance, except Stroop Colour and Interference where lower scores imply better performance.
UM-PDHQ: University of Miami Parkinson's disease Hallucinations Questionnaire: higher scores indicate more severe hallucinations.

Supplementary Table 2.

Longitudinal differences in white matter of thalamic tracts (mean fibre cross-section (FC)) between patients with Parkinson's disease and visual hallucinations (PD-VH) and without hallucinations (PD non VH).

Tract	Changes at Baseline			Visit 2 (18 months follow up)		
	beta	p-value	q-value	beta	p-value	q-value
**Left AV**	-0.0167	0.073575	0.176	**-0.159**	**0.005**	**0.006098**
**Left CeM**	-0.01496	0.106572	0.176	**-0.168**	**0.004**	**0.006098**
**Left CM**	-0.01536	0.087835	0.176	**-0.167**	**0.004**	**0.006098**
**Left L_Sg**	-0.0143	0.151725	0.217	**-0.157**	**0.005**	**0.006098**
**Left LD**	-0.01729	0.078379	0.176	**-0.162**	**0.005**	**0.006098**
**Left LGN**	-0.01614	0.100528	0.176	**-0.158**	**0.007**	**0.007609**
**Left LP**	-0.00259	0.825082	0.855	**-0.174**	**0.001**	**0.006098**
**Left MDl**	-0.00856	0.442954	0.481	**-0.146**	**0.002**	**0.006098**
**Left MDm**	-0.01157	0.261344	0.297	**-0.148**	**0.004**	**0.006098**
**Left MGN**	-0.01538	0.092495	0.176	**-0.165**	**0.004**	**0.006098**
**Left MVRe**	-0.01651	0.064117	0.176	**-0.167**	**0.004**	**0.006098**
**Left Pf**	-0.01552	0.065164	0.176	**-0.167**	**0.004**	**0.006098**
**Left PuA**	-0.00886	0.838129	0.855	**-0.131**	**0.027**	**0.028125**
**Left PuI**	-0.01694	0.076763	0.176	**-0.164**	**0.005**	**0.006098**
**Left PuL**	-0.0134	0.160321	0.217	**-0.166**	**0.005**	**0.006098**
**Left PuM**	-0.01653	0.106226	0.176	**-0.16**	**0.006**	**0.006818**
**Left VA**	-0.01349	0.190176	0.241	**-0.162**	**0.005**	**0.006098**
**Left VAmc**	-0.01667	0.070042	0.176	**-0.166**	**0.004**	**0.006098**
**Left VLa**	-0.01213	0.197567	0.241	**-0.168**	**0.004**	**0.006098**
**Left VLp**	-0.012	0.217166	0.253	**-0.165**	**0.004**	**0.006098**
**Left VPL**	-0.01255	0.15604	0.217	**-0.17**	**0.004**	**0.006098**
**Left VM**	0.054166	0.167992	0.221	**0.683**	**0.004**	**0.006098**
**Left CL**	-0.01582	0.104575	0.176	**-0.158**	**0.005**	**0.006098**
**Left Pc**	0.046429	0.071154	0.176	0.493	0.041	0.060981
**Left Pt**	0.046429	0.071154	0.176	0.072	0.086	0.087755
**Right AV**	-0.02101	0.020802	0.176	**-0.158**	**0.005**	**0.006098**
**Right CeM**	**-0.01569**	**9.85E-06**	**<0.001**	**-0.169**	**0.003**	**0.006098**
**Right CM**	-0.01791	0.041074	0.176	**-0.166**	**0.004**	**0.006098**
**Right L_S**	-0.02014	0.051005	0.176	**-0.159**	**0.006**	**0.006818**
**Right LD**	-0.02256	0.016982	0.176	**-0.16**	**0.005**	**0.006098**
**Right LGN**	-0.01993	0.107471	0.176	**-0.154**	**0.007**	**0.007609**
**Right LP**	-0.01586	0.110677	0.176	**-0.158**	**0.005**	**0.006098**
**Right MDl**	0.013326	0.432889	0.481	**-0.128**	**0.005**	**0.006098**
**Right MDm**	-0.03742	0.002003	0.05	**-0.152**	**0.004**	**0.006098**
**Right MGN**	-0.02135	0.112517	0.176	**-0.16**	**0.005**	**0.006098**
**Right MVRe**	-0.01951	0.032731	0.176	**-0.163**	**0.004**	**0.006098**

**Right Pf**	-0.015	0.088	0.176	**-0.169**	**0.003**	**0.006098**
**Right PuA**	-0.0088	0.520089	0.553	**-0.154**	**0.005**	**0.006098**
**Right PuI**	-0.02137	0.043955	0.176	**-0.162**	**0.005**	**0.006098**
**Right PuL**	-0.01752	0.061791	0.176	**-0.165**	**0.004**	**0.006098**
**Right PuM**	-0.02221	0.020873	0.176	**-0.158**	**0.006**	**0.006818**
**Right VA**	-0.01884	0.062591	0.176	**-0.161**	**0.005**	**0.006098**
**Right VAmc**	-0.02079	0.032996	0.176	**-0.161**	**0.005**	**0.006098**
**Right VLa**	-0.01488	0.11635	0.176	**-0.167**	**0.004**	**0.006098**
**Right VLp**	-0.01399	0.12015	0.177	**-0.169**	**0.003**	**0.006098**
**Right VPL**	-0.01395	0.079667	0.176	**-0.17**	**0.003**	**0.006098**
**Right VM**	0.063079	0.090592	0.176	**0.676**	**0.004**	**0.006098**
**Right CL**	-0.00386	0.86107	0.861	**-0.167**	**0.008**	**0.008511**
**Right Pc**	0.037085	0.202579	0.241	0.503	0.050	0.06098
**Right Pt**	0.037085	0.202579	0.241	0.052	0.119	0.119

AV*: anteroventral, CeM: central medial, CM: centromedian, L_Sg: limitans, LD: laterodorsal, LGN: lateral geniculate, LP: lateral posterior, MDl: mediodorsal medial parvocellular, MDm: mediodorsal medial magnocellular, MGN: medial geniculate, MVRe: Reuniens medial ventral, Pf: parafascicular, PuA: pulvinar anterior, PuI: pulvinar inferior, PuL: pulvinar lateral, PuM: pulvinar medial, VA: ventral anterior, VAmc: ventral anterior magnocellular, VLa: ventral lateral anterior, VLp: ventral lateral posterior, VPL: ventral posterolateral, VM: ventromedial, CL: central lateral, Pc: paracentral, Pt: paratenial.*

q: FDR-corrected p-value.

In bold tracts showing statistically significant differences between PD-VH and PD non VH participants after correction for multiple comparisons.

Effect of anxiety and depression

Given the observed differences in anxiety and depression between patients with Parkinson's disease (PD) with visual hallucinations (PD-VH) and without hallucinations (PD non VH) we performed additional analyses to ensure the observed structural white and grey matter changes were not directly influenced by anxiety and depression scores, assessed using the Hospital Anxiety and Depression Scale (HADS)[17].

We did not find any statistically significant correlation between HADS depression and anxiety scores and the main structural grey and white matter changes seen in PD-VH: specifically the mean volume of the medial mediodorsal magnocellular thalamic subnucleus (MDm) or mean fibre cross section (FC) of thalamic white matter tracts, either at baseline or longitudinally (Supplementary Table 1).

Supplementary table 3. Relationship between anxiety and depression scores and thalamic volumes and white matter tract integrity

	**Relationship with mean MDm volume**		**Relationship with mean tract FC**	
	**Baseline**	**Longitudinal difference**	**Baseline**	**Longitudinal difference**
**HADS anxiety**	rho=0.009 p=0.939	rho=0.036 p=0.758	rho = -0.111 p=0.338	rho = -0.100 p=0.388
**HADS depression**	rho=-0.102 p=0.381	rho=0.003 p=0983	rho=0.020 p=0.863	rho=-0.172 p=0.138

HADS: Hospital anxiety and depression questionnaire, higher scores indicate higher severity of anxiety or depression respectively. MDm: medial mediodorsal magnocellular thalamic subnucleus. FC: fibre cross section

Since HADS anxiety and depression scores were not correlated with our main outcome measures (MDm volume and tract FC) and they were inherently different in our compared groups (PD-VH versus PD non VH) we chose not to include them as covariates, as this could would reduce the sensitivity of our model to detect a true effect without improving specificity [20,21].

Supplementary Figure 2.

Correlation between HADS anxiety score and right mediodorsal medial magnocellular thalamic nucleus volume and white matter fibre cross section of thalamic tracts.

Change in thalamic nuclei volumes for the right mediodorsal medial magnocellular thalamic nucleus volume (MDm) at baseline (**A**) and after 18 months (**C**) and mean fibre cross-section (FC) for thalamic tracts at baseline (**B**) and at 18 months (**D**) in PD participants was correlated with severity of anxiety, assessed using the Hospital anxiety and depression questionnaire (HADS); higher scores indicate higher severity of anxiety. There were no statistically significant correlations between depression scores and volume or FC.

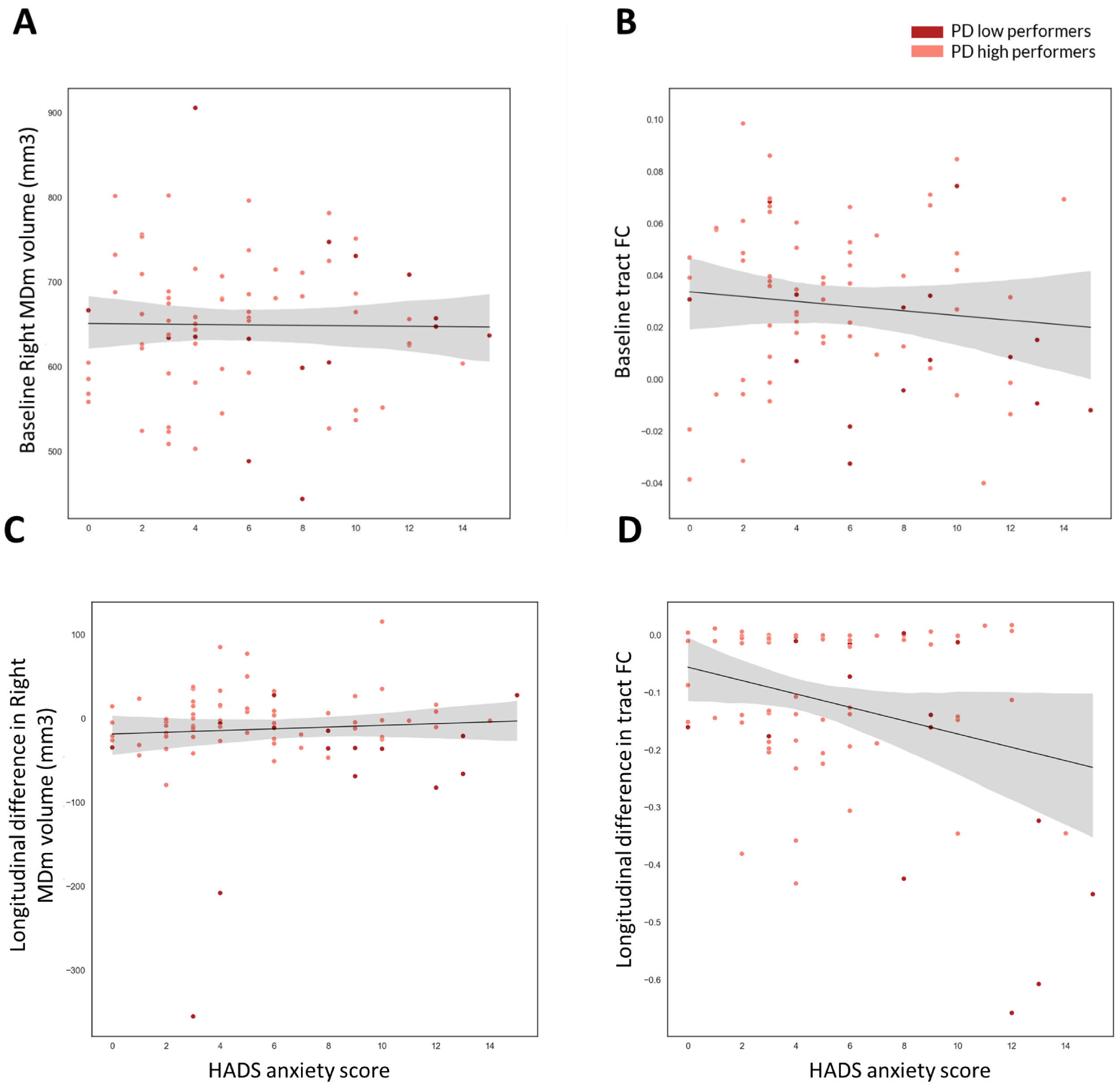

Supplementary Figure 3.

Correlation between HADS depression score and right mediodorsal medial magnocellular thalamic nucleus volume and white matter fibre cross section of thalamic tracts.

Change in thalamic nuclei volumes for the right mediodorsal medial magnocellular thalamic nucleus volume (MDm) at baseline (**A**) and after 18 months (**C**) and mean fibre cross-section (FC) for thalamic tracts at baseline (**B**) and at 18 months (**D**) in PD participants was correlated with severity of depression, assessed using the Hospital anxiety and depression questionnaire (HADS); higher scores indicate higher severity of anxiety. There were no statistically significant correlations between depression scores and volume or FC.

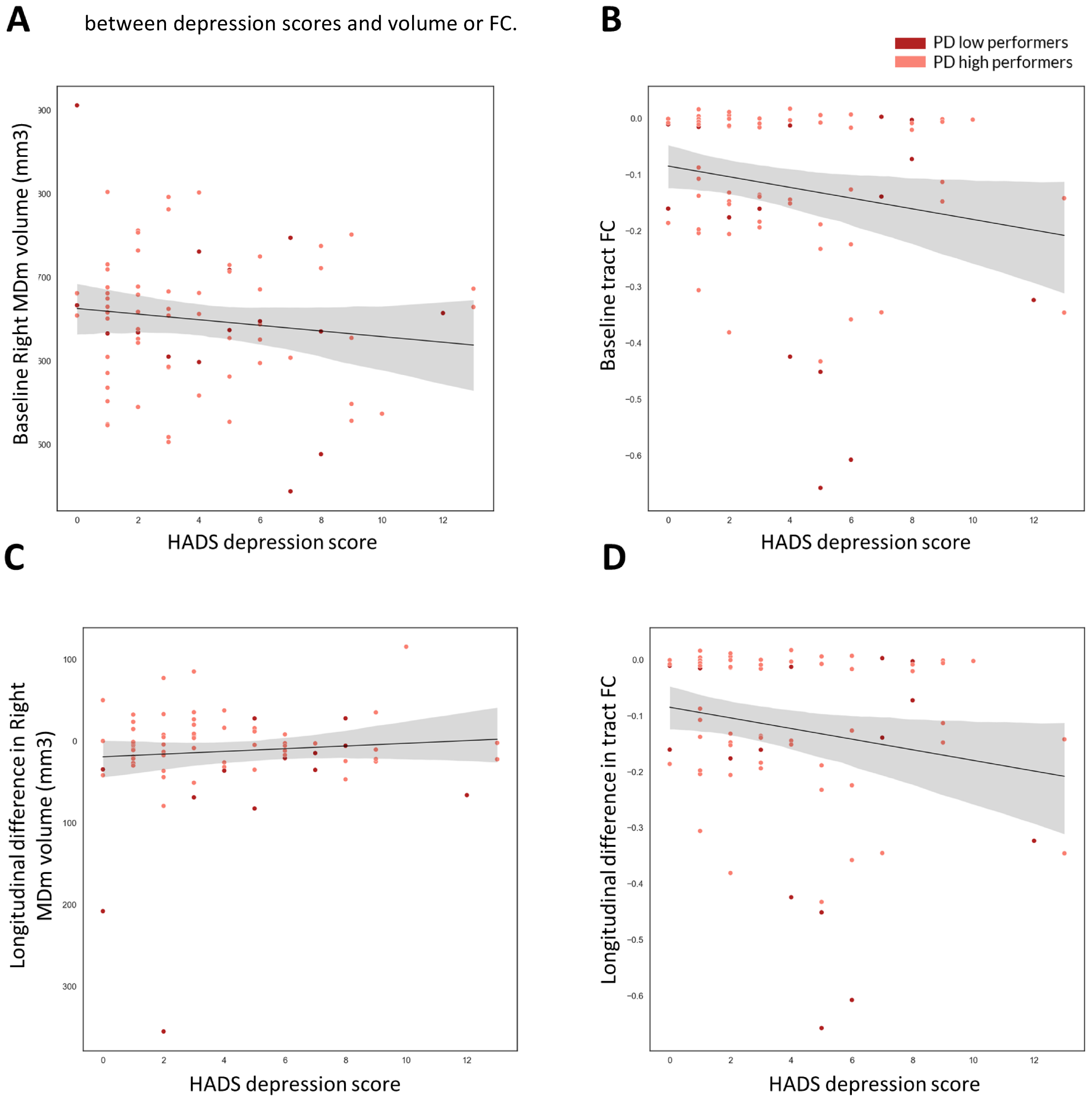

References

1 Roalf DR, Quarmley M, Elliott MA, *et al.* The impact of quality assurance assessment on diffusion tensor imaging outcomes in a large-scale population-based cohort. *NeuroImage* 2016;**125**:903–19. doi:10.1016/j.neuroimage.2015.10.068

2 Daniel SE, Lees AJ. Parkinson's Disease Society Brain Bank, London: overview and research. *J Neural Transm Suppl* 1993;**39**:165–72.

3 Creavin ST, Wisniewski S, Noel-Storr AH, *et al.* Mini-Mental State Examination (MMSE) for the detection of dementia in clinically unevaluated people aged 65 and over in community and primary care populations. *Cochrane Database Syst Rev* Published Online First: 2016. doi:10.1002/14651858.CD011145.pub2

4 Dalrymple-Alford JC, MacAskill MR, Nakas CT, *et al.* The MoCA: well-suited screen for cognitive impairment in Parkinson disease. *Neurology* 2010;**75**:1717–25. doi:10.1212/WNL.0b013e3181fc29c9

5 Wechsler, D. *Wechsler Adult Intelligence Scale-Fourth Edition*. Fourth. San Antonio, TX: : NCS Pearson 2008. https://www.pearsonclinical.com/psychology/products/100000392/wechsler-adult-intelligence-scalefourth-edition-wais-iv.html (accessed 6 Feb 2019).

6 Stroop JR. Studies of interference in serial verbal reactions. *J Exp Psychol* 1935;**18**:643–62. doi:10.1037/h0054651

7 Rende B, Ramsberger G, Miyake A. Commonalities and differences in the working memory components underlying letter and category fluency tasks: a dual-task investigation. *Neuropsychology* 2002;**16**:309–21.

8 Warrington EK. *Recognition Memory Test: Manual*. Berkshire: : UKNFER-Nelson 1984.

9 Warrington EK. The Graded Naming Test: A Restandardisation. *Neuropsychol Rehabil* 1997;**7**:143–6. doi:10.1080/713755528

10 Benton AL, Varney NR, Hamsher K deS. Visuospatial Judgment: A Clinical Test. *Arch Neurol* 1978;**35**:364–7. doi:10.1001/archneur.1978.00500300038006

11 Hooper H. *Hooper Visual Organization Test (VOT) Manual.* Los Angeles: : CA: Western Psychological Services 1983.

12 Sloan LL. New test Charts for the Measurement of Visual Acuity at far and Near Distances *. *Am J Ophthalmol* 1959;**48**:807–13. doi:10.1016/0002-9394(59)90626-9

13 Farnsworth Dean. *The Farnsworth dichotomous test for color blindness, panel D-15: manual*. New York: : Psychological Corp. 1947. https://catalog.hathitrust.org/Record/102201930 (accessed 23 Jan 2019).

14 Pelli D, Robson JG, Wilkins AJ. The design of a new letter chart for measuring contrast sensitivity. *Clin Vis Sci* 1988;**2**:187–99.

15 Goetz CG, Tilley BC, Shaftman SR, *et al.* Movement Disorder Society-sponsored revision of the Unified Parkinson's Disease Rating Scale (MDS-UPDRS): scale presentation and clinimetric testing results. *Mov Disord Off J Mov Disord Soc* 2008;**23**:2129–70. doi:10.1002/mds.22340

16 Hummel T, Sekinger B, Wolf SR, *et al.* “Sniffin” sticks’: olfactory performance assessed by the combined testing of odor identification, odor discrimination and olfactory threshold. *Chem Senses* 1997;**22**:39–52.

17 Zigmond AS, Snaith RP. The hospital anxiety and depression scale. *Acta Psychiatr Scand* 1983;**67**:361–70.

18 Stiasny-Kolster K, Mayer G, Schäfer S, *et al.* The REM sleep behavior disorder screening questionnaire--a new diagnostic instrument. *Mov Disord Off J Mov Disord Soc* 2007;**22**:2386–93. doi:10.1002/mds.21740

19 Tomlinson CL, Stowe R, Patel S, *et al.* Systematic review of levodopa dose equivalency reporting in Parkinson’s disease. *Mov Disord* 2010;**25**:2649–53. doi:10.1002/mds.23429

20 Miller GA, Chapman JP. Misunderstanding analysis of covariance. *J Abnorm Psychol* 2001;**110**:40–8. doi:10.1037/0021-843X.110.1.40

21 Rohrer JM. Thinking Clearly About Correlations and Causation: Graphical Causal Models for Observational Data. *Adv Methods Pract Psychol Sci* 2018;**1**:27–42. doi:10.1177/2515245917745629
